# Bridging the gap between economic regulation and the right to health: a study on renewable energy, financial inclusion, and under-5 mortality in EU administrative contexts

**DOI:** 10.3389/fpubh.2026.1812684

**Published:** 2026-04-24

**Authors:** Xuchun Ning, Rimsha Arshad

**Affiliations:** 1 Faculty of Law, Xiangtan University, Xiangtan, China; 2 Department of Economics, Guangdong University of Science and Technology, Dongguan, China

**Keywords:** domestic credit to private sector, forest area, income inequality, mortality rate under-5, research and development expenditure

## Abstract

This study examines the association between environmental sustainability factors and Domestic Credit to Private Sector (CPS) across selected EU countries, specifically focusing on Germany, France, Italy, Spain, Netherlands, Sweden, Finland, and Denmark. Using advanced econometric techniques, including MMQR, the study investigates how renewable energy consumption (REC), research and development expenditure (RDE), income inequality (GIN), and under-5 mortality (MUR) influence CPS. The results show that CPS is significantly impacted by REC and RDE, suggesting that financial access is closely tied to investments in renewable energy and technological innovation. Furthermore, CPS is negatively associated with income inequality, highlighting the potential of financial systems to reduce disparities and promote more inclusive economic growth. Although CPS is positively associated with forest area (FAR), the relationship with under-5 mortality is limited, implying that healthcare infrastructure and social policies play a more direct role in improving child health outcomes. These findings emphasize the importance of integrating green finance policies, enhancing R&D investments, and addressing income inequality to foster long-term sustainable development and financial inclusion in EU countries.

## 1 Introduction

Sustainability has become a central focus for governments, businesses, and institutions worldwide, as it serves as the cornerstone for ensuring long-term prosperity, environmental protection, and social equity. The selected European development with its collective ambition to lead the world in achieving sustainable development, has made significant strides in integrating environmental, economic, and social sustainability into its policy frameworks. Key to this effort are the selected EU’s commitments to achieving the Sustainable Development Goals (SDGs), which underscore the importance of reducing inequalities, enhancing economic growth, addressing environmental challenges, and improving public health outcomes (1). Among these, financial mechanisms such as Domestic Credit to Private Sector (% of GDP) have emerged as a critical enabler of sustainable growth, facilitating investments in key sectors like renewable energy, innovation, and social welfare (2).

Within the selected EU, the role of financial systems in promoting sustainability is increasingly recognized, but the specific mechanisms through which Domestic Credit to Private Sector is associated with sustainability outcomes remain inadequately explored (3). As the private sector plays a pivotal role in driving green transitions and technological innovation, understanding how financial access is associated with sustainability in various dimensions becomes essential. Credit availability enables firms to invest in renewable energy, expand green technologies, and contribute to reducing carbon emissions, aligning with the selected EU’s ambitious goal of achieving climate neutrality by 2050 (4). The transition to renewable energy, for example, is a critical pathway for reducing reliance on fossil fuels and combating climate change. The selected EU’s efforts to increase the Renewable Energy Share of Total Energy Consumption are significant, with several member states already making substantial progress in clean energy adoption. However, achieving these goals requires substantial investment. Access to Domestic Credit to Private Sector is fundamental for facilitating investments in energy infrastructure, technological advancements, and other green initiatives that contribute to a sustainable energy future (5).

The importance of Research & Development (R&D) Expenditure in fostering innovation cannot be overstated. R&D investments are integral to developing new technologies that can address environmental challenges, improve energy efficiency, and accelerate the adoption of sustainable practices (6). By enabling increased R&D funding, domestic credit plays a crucial role in fostering innovation and driving the technological advancements required for achieving long-term sustainability. In addition to economic and environmental factors, social sustainability is equally important (7). Income inequality, as measured by the Gini Index, remains a significant issue within many selected EU countries, affecting social cohesion and overall well-being. Unequal access to resources and opportunities can undermine sustainability efforts, as it hinders inclusive development (8). Understanding the relationship between domestic credit and income inequality can offer valuable insights into how financial systems can be leveraged to reduce disparities and promote more equitable outcomes (9).

Another essential aspect of sustainability is the preservation of natural resources. Forest Area is a key indicator of ecological sustainability, as forests contribute to carbon sequestration, biodiversity conservation, and the overall health of ecosystems. Selected EU policies aimed at reducing deforestation and promoting sustainable land management practices rely heavily on investments in land restoration, afforestation, and conservation. The availability of domestic credit is crucial for financing these efforts, which directly contribute to achieving environmental sustainability goals (10). Furthermore, the Mortality Rate Under-5 is an important social indicator that reflects both the healthcare system’s effectiveness and broader socio-economic conditions. High child mortality rates are often associated with poverty, inadequate healthcare infrastructure, and poor living conditions. By facilitating investments in healthcare infrastructure and improving access to resources, domestic credit can play an indirect yet vital role in enhancing child health outcomes, thereby contributing to social sustainability (11). This study uses precise indicators for financial access, renewable energy consumption, and environmental sustainability to explore their association with child health outcomes. These variables are defined and measured as follows: financial access through bank account ownership and access to credit, renewable energy as a share of total energy consumption, and environmental sustainability through CO2 emissions per capita.

**Figure 1:**
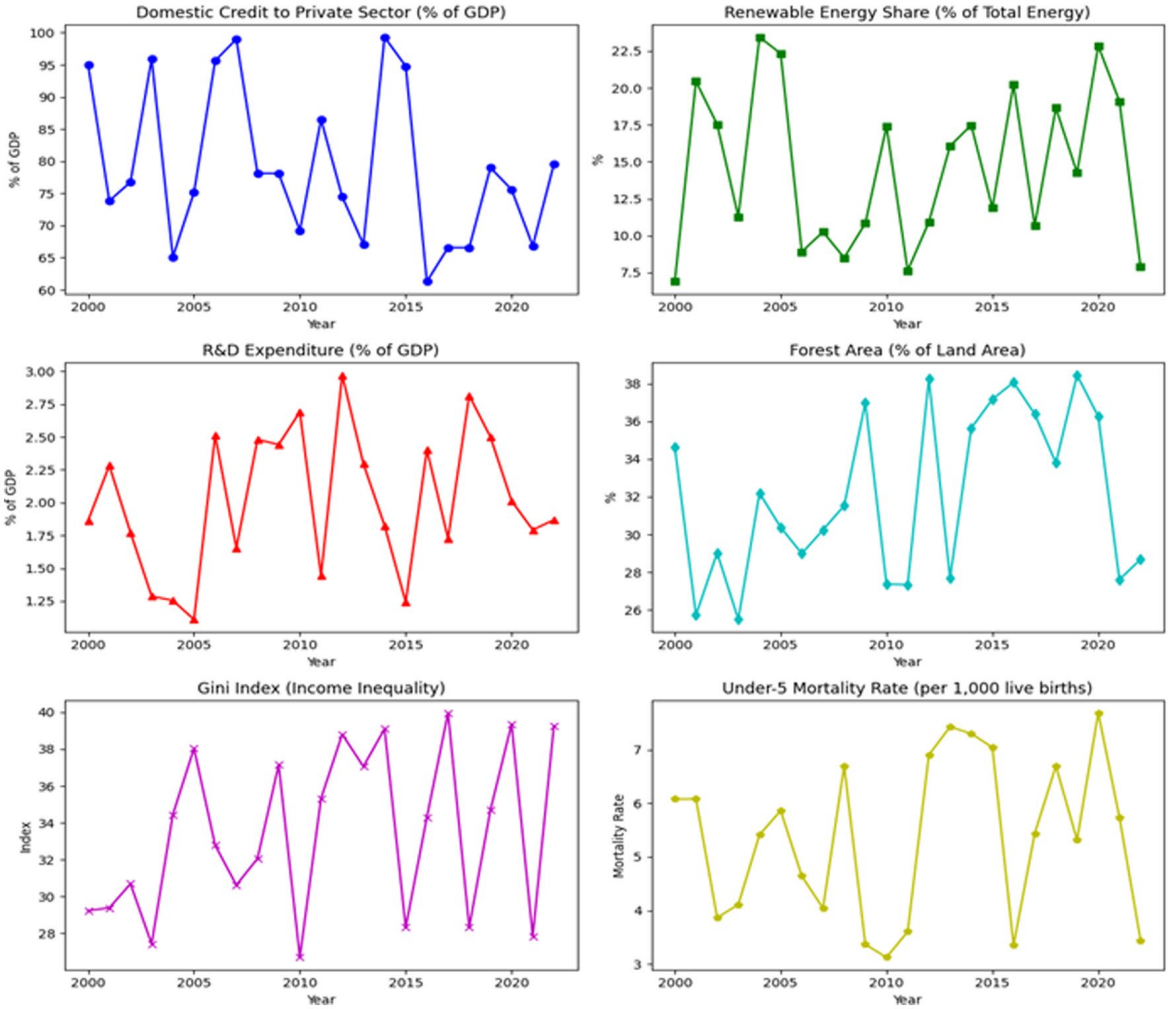
Trends of key sustainability variables for selected EU countries (2000–2022).

Figure 1 illustrates the trends of key sustainability variables for selected EU countries from 2000 to 2022. Over this period, Domestic Credit to Private Sector has shown gradual growth, indicating increasing financial access for private sector development. The Renewable Energy Share has steadily risen, reflecting the selected EU’s commitment to transitioning to cleaner energy sources, though with significant variation across countries. R&D Expenditure fluctuates within a narrow range, underlining the selected EU’s ongoing investment in technological innovation, particularly in green technologies. The Forest Area has remained relatively stable, with some countries showing a slight increase, signaling the importance of environmental conservation efforts. Income Inequality, as measured by the Gini Index, remains a concern, with some countries experiencing widening gaps, while others show marginal improvements. Lastly, the Under-5 Mortality Rate has generally declined, reflecting improvements in healthcare systems across the selected EU. Together, these trends highlight the complex interplay between financial systems, environmental policies, technological innovation, and social equity in advancing sustainability goals.

The primary objective of this study is to examine how Domestic Credit to Private Sector (% of GDP) is associated with various sustainability outcomes in the selected EU context. Specifically, this research aims to assess the relationships of domestic credit on Renewable Energy Share, R&D Expenditure, Income Inequality, Forest Area, and Under-5 Mortality Rate. By exploring these relationships, the study seeks to provide empirical evidence on the role of financial access in driving both environmental and social sustainability (12). This study focuses on a selected group of EU countries, namely Germany, France, Italy, Spain, Netherlands, Sweden, Finland, and Denmark, due to their diverse economic profiles, varying stages of green growth, and robust data availability. These countries represent a cross-section of the EU’s broader economic landscape, making them ideal for examining how Domestic Credit to Private Sector is associated with sustainability outcomes. Their ongoing efforts in renewable energy adoption, technological innovation, and social equity provide a rich context for understanding the role of financial systems in advancing the EU’s sustainability agenda. The selection of these countries allows for an analysis that reflects both economic diversity and policy relevance within the EU.

The study contributes to existing literature in several important ways. Financials on Domestic Credit to Private Sector as the central independent variable, addressing a gap in the literature where financial systems are often studied in isolation without explicitly linking them to specific sustainability outcomes. This research examines how domestic credit not only facilitates economic growth but also promotes environmental sustainability, technological innovation, and social equity. The study utilizes Method of Moments Quantile Regression (MMQR) to capture the heterogeneous estimated associations of credit on sustainability across different quantiles. This methodological approach allows for a more nuanced understanding of how financial access associations countries at varying levels of development, considering both the best-performing and the least-performing countries in the selected EU (13). By examining these relationships across multiple quantiles, the study offers a comprehensive view of how domestic credit is associated with sustainability outcomes under different economic conditions (14). This study investigates the role of financial access and renewable energy adoption in improving child health outcomes, particularly under-5 mortality. While financial access may enhance healthcare affordability, maternal care, nutrition, and sanitation, renewable energy adoption may contribute by reducing pollution and improving indoor air quality. This paper will articulate the conceptual mechanisms linking these factors to health outcomes and propose a causal framework.

The methodological contribution of this research lies in its use of advanced econometric techniques to analyze panel data across selected EU countries. By employing OLS, HAC, and Cluster-Robust estimation methods, the study ensures robustness in its findings, addressing potential biases and heteroskedasticity in the data. This comprehensive approach strengthens the reliability of the results and provides policymakers with actionable insights.

Furthermore, the study’s findings offer valuable policy contributions. By highlighting the critical role of financial access in promoting renewable energy, reducing inequality, fostering innovation, and improving social outcomes, the study provides guidance on how selected EU financial systems can be better leveraged to support sustainable development (15). It also contributes to the broader discussion on how financial mechanisms can be aligned with environmental and social goals to ensure long-term sustainability.

In conclusion, this research aims to provide a deeper understanding of the interplay between financial systems and sustainability outcomes in the selected EU, offering evidence-based insights that can inform future policies and investment strategies. By focusing on Domestic Credit to Private Sector as the central driver of sustainability, the study contributes to both academic literature and practical policy discussions on sustainable finance and development.

## 2 Literature review

The concept of sustainability has garnered significant attention in global academic and policy circles, especially in the context of achieving the United Nations Sustainable Development Goals (SDGs). Among the selected EU’s primary sustainability objectives are reducing environmental associations, enhancing social welfare, and promoting economic growth. The relationship between financial access, particularly Domestic Credit to Private Sector (% of GDP), and sustainability outcomes has gained substantial recognition in literature. Financial systems play a pivotal role in supporting sustainable growth by directing investments into sectors such as renewable energy, technological innovation, healthcare, and infrastructure development (16). This section synthesizes key studies in the field, focusing on the connection between financial access and key sustainability indicators, including renewable energy, income inequality, innovation (R&D), forest preservation, and public health outcomes.

### 2.1 Domestic credit to private sector and economic growth

The relationship between financial systems and economic growth is well-documented. Early works such as Becker and Knudsen (17) emphasized the role of finance in fostering entrepreneurship, while Beck et al. (18) established that access to credit enables economic growth by facilitating investments, job creation, and innovation. More recent studies, like those of Levine (19), confirm that financial systems help optimize the allocation of resources, contributing to higher productivity and sustained economic development. In the context of the selected EU, financial systems, particularly Domestic Credit to Private Sector, are seen as a key driver of private sector development. Research by Casu et al. (20) finds that access to credit positively associations firm growth, which in turn stimulates economic growth. However, the selected EU’s progress in sustainable economic growth requires a deeper understanding of how financial access facilitates green and inclusive growth, making it a vital area of exploration in this study.

### 2.2 Domestic credit and renewable energy transition

The selected EU’s renewable energy goals are critical to achieving long-term sustainability, aiming to reduce carbon emissions and transition to cleaner energy sources. However, financing the transition to renewable energy is a significant challenge. Studies such as Kathuria et al. (21) and Jepson et al. (22) highlight those financial systems, including Domestic Credit to Private Sector, are essential for enabling investments in green technologies. According to Pérez-López et al. (23), the role of financial markets in supporting renewable energy projects is indispensable in countries where public sector investment is insufficient to meet climate goals. Moreover, Hettinga et al. (24) emphasize that domestic credit can unlock private sector funding for renewable energy infrastructure, which is crucial for meeting the selected EU’s renewable energy targets. This study seeks to extend these findings by specifically examining the relationship between financial access and the share of renewable energy in selected EU countries, investigating whether domestic credit directly correlates with increased renewable energy adoption.

### 2.3 R&D expenditure and sustainable innovation

Innovation is critical for addressing sustainability challenges, and Research and Development (R&D) is the primary vehicle for driving technological advancements. The selected EU’s commitment to innovation is underscored by the Pollex and Lenschow (25) framework, which funds R&D initiatives aimed at tackling environmental and energy-related challenges. Aghion et al. (26) and Slobodyan (27) provide foundational work showing that R&D investment is essential for long-term economic and technological development. Furthermore, Mazzucato and Robinson (28) underscores the role of public-private collaboration in driving sustainable innovations, especially in clean technologies. In the selected EU context, R&D expenditure is often constrained by limited access to funding, particularly for smaller firms. As noted by Federico et al. (29), access to Domestic Credit to Private Sector is crucial for increasing R&D investments, which can accelerate the development of green technologies. This study contributes by analyzing how financial access is associated with R&D expenditure specifically for sustainable innovation in selected EU countries.

### 2.4 Domestic credit and income inequality

Income inequality remains a pressing concern in many selected EU countries, with significant implications for social stability and sustainable development. Brei et al. (30) have argued that financial systems can either exacerbate or reduce income inequality. Domestic Credit to Private Sector plays a crucial role in this dynamic. On the one hand, access to credit can empower individuals and businesses, particularly those in disadvantaged regions, by providing opportunities for entrepreneurship and economic mobility. On the other hand, unequal access to credit can deepen wealth disparities. Studies by Beck et al. (31) and Ranaldi and Milanović (32) suggest that inclusive financial systems that provide broader access to credit can mitigate inequality. In the selected EU context, financial inclusion and its association with reducing income inequality remain a crucial area for investigation. This study will explore the relationship between Domestic Credit to Private Sector and income inequality across selected EU countries, assessing whether financial access can contribute to more equitable growth.

### 2.5 Forest preservation and sustainable land use

Sustainable land use and forest preservation are integral to achieving the selected EU’s environmental sustainability goals. Forests play a critical role in carbon sequestration and maintaining biodiversity. The selected EU Forest Strategy and initiatives like the Forest Carbon Partnership Facility (FCPF) emphasize the need for increased investment in forest conservation. However, achieving these goals requires substantial financing. Schirmer and Bull (33) argue that financial resources, including domestic credit, are necessary to fund reforestation and afforestation projects, as well as sustainable land management. The role of Domestic Credit to Private Sector in financing forest-related projects has been underexplored, particularly in the selected EU context. This study aims to fill this gap by examining how credit systems can support sustainable forest management and conservation efforts in selected EU countries.

### 2.6 Domestic credit and health outcomes

The mortality rate under-5 is a key indicator of public health and social development. Access to financial resources is directly linked to the availability and quality of healthcare services. Koç et al. (34) argue that economic growth, facilitated by access to credit, plays a significant role in improving healthcare systems. In the selected EU, high-quality healthcare systems contribute to low mortality rates, particularly for children. However, disparities in access to healthcare remain, especially in lower-income regions. By increasing Domestic Credit to Private Sector, governments can enhance healthcare infrastructure and reduce health inequalities. Barber and West (35) highlights the role of financial access in improving public health outcomes, showing that investments in healthcare infrastructure are essential for achieving long-term sustainability. This study seeks to analyze how domestic credit is associated with child health outcomes across selected EU countries. From an environmental health perspective, child mortality is closely linked to exposure to environmental risk factors, particularly air pollution and climate-related stressors. Evidence from environmental epidemiology shows that exposure to fine particulate matter (PM2.5), poor air quality, and household or ambient pollution significantly increases the risk of respiratory infections, low birth weight, and infant mortality. In this context, transitions toward renewable energy and improved environmental conditions can reduce pollution exposure pathways, thereby contributing to better child health outcomes. Additionally, climate-related factors such as heat stress and environmental degradation can exacerbate health vulnerabilities among children, particularly in urban and densely populated areas. These mechanisms highlight the indirect but important role of environmental sustainability in shaping public health outcomes, reinforcing the relevance of integrating environmental and financial dimensions in the analysis. In environmental epidemiology, these pathways are well established, where exposure to ambient and household air pollution is directly linked to respiratory infections, impaired lung development, and increased child mortality. Children are particularly vulnerable due to their developing immune systems and higher exposure relative to body weight. In addition, climate-related risks such as heat stress, extreme weather events, and environmental degradation can indirectly affect child health by disrupting food systems, water quality, and access to healthcare services. These mechanisms highlight the importance of integrating environmental exposure pathways into sustainability and public health analysis.

### 2.7 Research gaps and future directions

While significant literature exists on the role of financial systems in driving sustainable development, several gaps remain, particularly in the context of the selected EU. First, he relationship between Domestic Credit to Private Sector and key sustainability outcomes, such as renewable energy adoption, R&D expenditure, and social equity, remains underexplored, especially when considering the nuanced estimated associations across different selected EU member states. Second, there is limited research on the heterogeneous estimated associations of financial access across quantiles of development, particularly in the selected EU’s diverse economic landscape (36). While the direct relationship of renewable energy consumption (REC) and forest area (FAR) on child health outcomes, such as Under-5 mortality (MUR), may not be immediately apparent, these variables are indirectly related to public health. Improved environmental quality through cleaner energy and forest conservation contributes to healthier living conditions, reducing pollution such as PM2.5, which is a known risk factor for infant and child health. Most studies focus on average relationships, overlooking the variation in outcomes across countries at different stages of development.

Future research should explore the interaction between domestic credit and other financial mechanisms such as green finance, Environmental, Social, and Governance (ESG) investments, and sustainable financial products. Moreover, further analysis is needed to understand how financial systems can be optimized to foster inclusive growth while supporting environmental sustainability, especially in the face of rising inequality within the selected EU (37). Lastly, more studies are needed to investigate the causal relationships between financial access and social outcomes, such as healthcare and education, to better inform policy interventions aimed at achieving the SDGs.

## 3 Theoretical or conceptual framework

This study aims to investigate the relationship between Domestic Credit to Private Sector (% of GDP) and various sustainability outcomes in the selected EU, specifically focusing on renewable energy adoption, R&D expenditure, income inequality, forest area, and child mortality rates. The conceptual framework integrates economic, financial, and sustainability theories to understand how financial access is associated with sustainable development, particularly in the context of the selected EU’s climate and social objectives.

### 3.1 Theoretical background

The theoretical foundation of this study is rooted in several key economic theories that emphasize the role of financial systems in fostering economic growth, technological innovation, and sustainability:

1. Financial development theory: Fengju and Wubishet (38) argue that financial systems facilitate economic growth by enabling efficient allocation of resources. The access to credit allows firms to invest in productive sectors, including green technologies and renewable energy. Ramírez Guerra (39) further emphasized that financial intermediation, through access to credit, is essential for fostering entrepreneurship and innovation, which are critical for achieving long-term sustainability.
2. Green growth theory: According to Liu et al. (40), economies can grow while reducing environmental associations, if there is a substantial investment in environmentally sustainable technologies and infrastructure. This theory is particularly relevant in the context of the selected EU, which is actively pursuing a green energy transition. Domestic Credit to Private Sector plays a key role in facilitating investments in renewable energy and green technologies, thus driving the green growth agenda.
3. Endogenous growth theory: Gabardo et al. (41) argues that technological innovation, supported by financial resources, is a key driver of economic growth. In the context of sustainability, this theory suggests that R&D expenditure in green technologies, fueled by access to finance, can lead to breakthroughs that reduce carbon emissions and enhance resource efficiency.
4. Income inequality and financial inclusion: Mdingi and Ho (42) have explored the dual role of financial systems in both exacerbating and reducing income inequality. Domestic Credit to Private Sector can reduce inequality by providing access to finance for marginalized groups, enabling economic mobility and fostering inclusive growth.
5. Sustainable development theory: As articulated by Costa-climent and Haftor (43) in the Brundtland Report, sustainability requires a balance between economic growth, environmental preservation, and social equity. Domestic Credit to Private Sector serves as a key enabler in achieving this balance, facilitating investments in all three pillars of sustainability.

### 3.2 Conceptual framework

The conceptual framework for this study is built on the assumption that Domestic Credit to Private Sector plays a pivotal role in influencing multiple sustainability outcomes. Financial inclusion associations with child health through several key channels. It is linked with improvements in healthcare affordability, ensures better access to maternal and preventive care, and enhances nutrition and sanitation conditions. On the other hand, renewable energy adoption has direct health benefits by reducing pollution, enhancing indoor air quality, and improving the reliability of electricity supply in healthcare facilities. The framework establishes the following relationships:

1 Domestic credit to private sector (independent variable):

o This variable is conceptualized as the central determinant of sustainable development in the selected EU, influencing various sectors such as renewable energy, innovation, social equity, and environmental preservation.

2 Dependent variables:

o Renewable energy share: Domestic Credit to Private Sector is hypothesized to positively association the adoption of renewable energy by facilitating investments in clean energy technologies and infrastructure.
o R&D Expenditure: Access to credit enables greater investment in R&D, particularly in green technologies and sustainable innovations.
o Income inequality (Gini Index): Increased access to financial resources can reduce inequality by promoting financial inclusion, facilitating entrepreneurship, and enabling access to capital for underserved populations.
o Forest area: Financial access supports sustainable land use practices, including afforestation and reforestation projects.
o Under-5 mortality rate: Domestic Credit to Private Sector can improve public health outcomes by enabling investments in healthcare infrastructure and services.

#### 3.2.1 Mechanisms of influence

- Green growth: Domestic Credit to Private Sector facilitates investments in renewable energy, clean technologies, and sustainable land management, contributing to green growth and environmental sustainability.
- Technological innovation: Access to credit enables firms to invest in R&D that fosters innovation in green technologies and energy-efficient solutions.
- Social equity: Credit access empowers marginalized groups and is linked with improvements in income distribution by providing financial resources for education, health, and entrepreneurship.

The conceptual framework further distinguishes between direct and indirect pathways through which financial access and environmental sustainability is associated with child health outcomes. Financial access (CPS) primarily operates through indirect socio-economic channels, including improved affordability of healthcare services, enhanced access to maternal and preventive care, better nutrition, and improved sanitation conditions. In contrast, environmental factors such as renewable energy consumption (REC) and forest areas (FAR) is associated with child health through both direct and indirect mechanisms. Direct pathways include reduced exposure to air pollution and improved indoor air quality, while indirect relationships arise through enhanced energy reliability for healthcare services and improved living conditions. These differentiated pathways highlight that the relationship between financial and environmental variables and under-5 mortality is mediated by broader socio-economic and environmental processes rather than representing immediate causal relationships.

**Figure 2:**
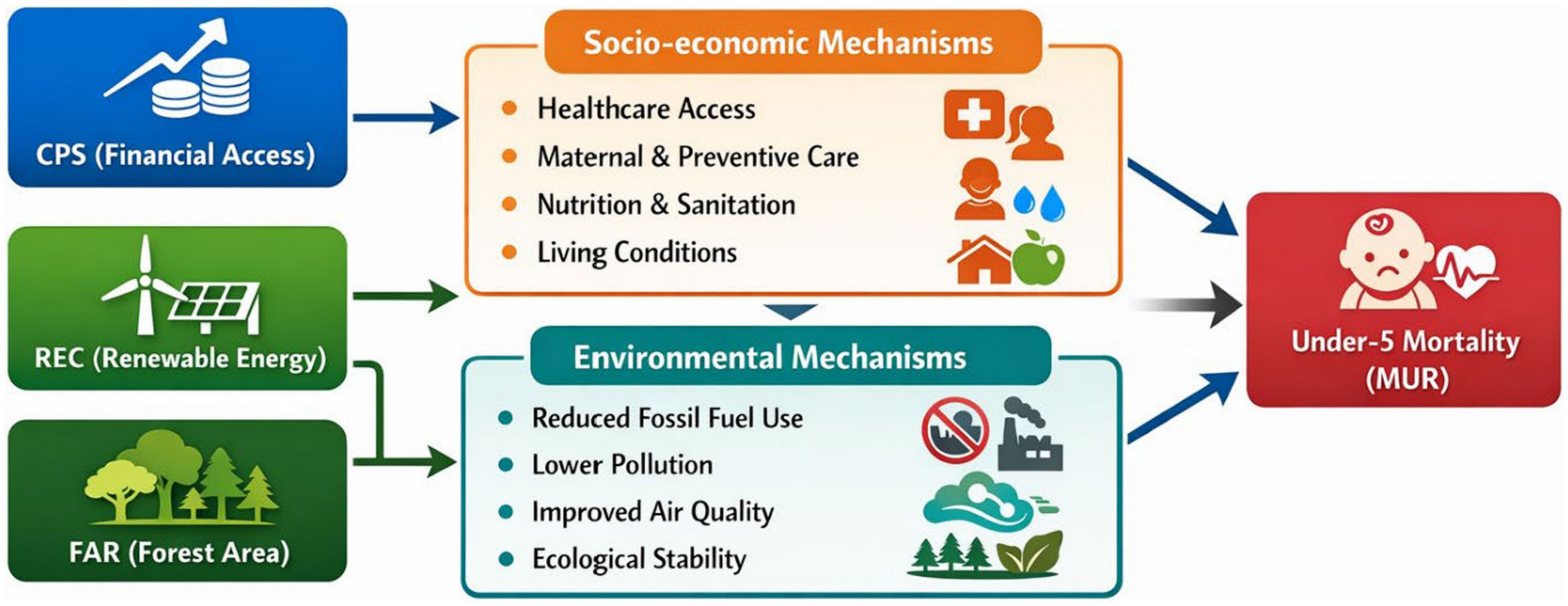
Conceptual framework linking financial access, environmental sustainability, and child health outcomes.

To improve clarity and theoretical coherence, the conceptual relationships proposed in this study are summarized in Figure 2. The framework distinguishes between socio-economic and environmental transmission mechanisms through which financial access and sustainability factors are associated with child health outcomes. Financial access (CPS) operates primarily through socio-economic pathways, including improved healthcare affordability, access to maternal and preventive services, and enhanced living conditions. In contrast, renewable energy consumption (REC) and forest areas (FAR) are associated with environmental pathways, particularly through reduced pollution exposure, improved air quality, and better environmental conditions. These pathways jointly influence under-5 mortality (MUR), highlighting that the relationship between financial systems, environmental sustainability, and child health is indirect and mediated through broader structural mechanisms rather than immediate causal effects.

To strengthen the environmental health dimension of the framework, it is important to clarify that Renewable Energy Consumption (REC) and Forest Area (FAR) do not represent direct measures of environmental exposure, such as particulate matter (PM2.5) or air quality indices. Instead, these variables are conceptualized as indirect structural proxies that influence child health outcomes through environmental exposure pathways. Specifically, higher renewable energy consumption is associated with reduced reliance on fossil fuels, leading to lower emissions of harmful pollutants, including fine particulate matter and other airborne toxins. Similarly, forest area contributes to environmental quality by acting as a natural carbon sink, improving air filtration, and mitigating local pollution levels. These mechanisms operate through reduced exposure to environmental health risks, particularly respiratory and pollution-related conditions that disproportionately affect children. Therefore, within this study, REC and FAR are integrated into the analytical framework as upstream environmental determinants that shape health outcomes indirectly via pollution exposure pathways rather than as direct measures of environmental quality.

Figure 2 illustrates a structured conceptual framework showing how financial access and environmental sustainability jointly influence child health outcomes through distinct transmission channels. Domestic Credit to Private Sector (CPS) operates through socio-economic mechanisms, including improved healthcare access, maternal and preventive care, better nutrition, and enhanced living conditions, which collectively contribute to reductions in under-5 mortality (MUR). In parallel, Renewable Energy Consumption (REC) and Forest Area (FAR) function as environmental determinants, influencing health outcomes through reduced reliance on fossil fuels, lower pollution levels, improved air quality, and greater ecological stability. These environmental pathways reduce exposure to harmful pollutants, indirectly supporting child health. The framework highlights that both financial and environmental factors do not exert direct effects on mortality but operate through mediated socio-economic and environmental processes, emphasizing the integrated and indirect nature of sustainability–health linkages.

### 3.3 Hypotheses of the study

Based on the theoretical and conceptual framework, the following hypotheses are formulated to guide the empirical analysis of the study:

- *H1*: Domestic Credit to Private Sector is positively associated with Renewable Energy Share in selected EU countries.
- *H2*: Domestic Credit to Private Sector is positively associated with R&D Expenditure in selected EU countries.
- *H3*: Domestic Credit to Private Sector reduces Income Inequality (Gini Index) in EU countries.
- *H4*: Domestic Credit to Private Sector is positively associated with Forest Area in selected EU countries.
- *H5*: Domestic Credit to Private Sector reduces Mortality Rate Under-5 in selected EU countries.

### 3.4 Research model

The research model for this study is grounded in the relationships outlined in the conceptual framework. The central independent variable, Domestic Credit to Private Sector, is hypothesized to is associated with various sustainability outcomes. The study will employ panel data analysis using Method of Moments Quantile Regression (MMQR) to capture the heterogeneous relationships of credit across different levels of development and sustainability performance.

The hypotheses will be tested using advanced econometric techniques to assess the direction, strength, and significance of the relationships between Domestic Credit to Private Sector and the dependent variables. This model will contribute to understanding how financial access can drive the green energy transition, reduce inequality, promote innovation, and improve public health outcomes in the selected EU.

## 4 Data and methodology

### 4.1 Data source and coverage

This study uses panel data from selected EU (Germany, France, Italy, Spain, Sweden, Netherlands, Denmark and Finland) countries spanning the years 2000 to 2022. The dataset spans from [2000–2022], depending on data availability and is drawn primarily from the World Development Indicators (WDI) published by the World Bank. WDI provides internationally comparable data on environmental, social, and financial dimensions, making it highly suitable for cross-country sustainability analysis.

### 4.2 Variable selection and measurement

The study considers a set of variables that represent the environmental, economic, and social dimensions of sustainability.

- Domestic Credit to Private Sector (% of GDP): Measures the depth of the financial system by indicating the proportion of domestic credit allocated to the private sector.
- Renewable Energy Share of Total Energy Consumption (%): The percentage of energy consumption derived from renewable sources like wind, solar, and hydropower.
- Research & Development Expenditure (% of GDP): The proportion of GDP spent on research and development, reflecting a country’s commitment to technological innovation and sustainability.
- Gini Index (Income Inequality): Measures income distribution, where 0 represents perfect equality and 100 represents extreme inequality.
- Forest Area (% of Land Area): The proportion of land area covered by forests, an important environmental sustainability indicator.
- Mortality Rate Under-5 (per 1,000 live births): Reflects health outcomes related to infant mortality and overall public health.

The selection of control variables is guided by the health production function and sustainable development framework, which emphasize that child health outcomes are shaped by interrelated economic, environmental, and social determinants. In this study, income inequality (GIN), renewable energy consumption (REC), forest area (FAR), and research and development expenditure (RDE) are included as key control variables, as they capture socio-economic disparity, environmental quality, ecological stability, and innovation capacity. Prior literature indicates that income inequality affects access to healthcare and essential services, while environmental conditions such as clean energy use and forest preservation are associated with improved living conditions and reduced exposure to harmful pollutants. R&D expenditure is also relevant as it reflects technological progress that may enhance healthcare systems and environmental management.

It is important to note that while environmental sustainability is proxied through REC and FAR, these variables do not directly measure pollution exposure. Instead, they capture broader environmental conditions that are associated with air quality and ecological health. In the absence of direct pollution indicators such as PM2.5, REC and FAR serve as indirect proxies reflecting structural environmental improvements that may reduce exposure to harmful pollutants. This distinction is critical for interpreting the results, as the estimated relationships represent indirect environmental health pathways rather than direct exposure–outcome effects.

However, child mortality is influenced by a broader set of determinants, including maternal education, healthcare quality, vaccination coverage, urbanization, and air pollution exposure. Due to data availability and consistency constraints across selected EU countries, these variables are not included in the empirical model, which may introduce potential omitted variable bias. This limitation is acknowledged and discussed in the limitations section.

Table 1 presents the descriptions for the variables used in this study. The Research and Development Expenditure (RDE) variable measures the percentage of GDP allocated to research and development efforts, which is expected to be associated with sustainability outcomes, particularly in technological innovation and green energy adoption. The Gini Index (GIN) represents income inequality within a country, serving as a critical social indicator of equity. CPS (Credit to the Private Sector) captures the level of financial resources allocated to private entities, influencing economic growth and access to sustainable finance. Forest Area (FAR) indicates the proportion of land covered by forests, a key environmental indicator linked to biodiversity and climate change mitigation. The Mortality Rate Under-5 (MUR) reflects child health outcomes, serving as an indicator of healthcare accessibility and overall socio-economic development. Lastly, REC (Renewable Energy Consumption) represents the proportion of total energy consumption derived from renewable sources, highlighting a country’s progress in achieving sustainability in energy consumption. All data is sourced from the World Bank’s World Development Indicators (WDI) dataset.

**Table 1:** Variable descriptions.

Variable	Abbreviation	Description	Data source
Research and development expenditure (% of GDP)	RDE	Percentage of GDP allocated to research and development	World Bank (WDI)
Gini index	GIN	Measures income inequality within a country	World Bank (WDI)
Domestic credit to private sector (% of GDP)	CPS	Ratio of credit given to the private sector as a percentage of GDP	World Bank (WDI)
Forest area (% of land area)	FAR	Percentage of land covered by forests	World Bank (WDI)
Mortality rate, under 5 (per 1,000 live births)	MUR	Number of deaths under the age of 5 per 1,000 live births	World Bank (WDI)
Renewable energy consumption (% of total final energy consumption)	REC	The share of renewable energy in total energy consumption	World Bank (WDI)

The variables used in this study are defined to capture the financial, environmental, and social dimensions of sustainability within a consistent empirical framework. Domestic Credit to Private Sector (CPS) is used as a proxy for financial access and reflects the overall financial depth of the economy, measured as the proportion of credit allocated to the private sector relative to GDP. It captures access to financial resources available for investment in healthcare, infrastructure, and social services that may indirectly influence maternal and child health outcomes. Renewable Energy Consumption (REC) represents the share of total final energy consumption derived from renewable sources such as wind, solar, and hydropower, reflecting the transition toward cleaner energy systems rather than energy production or installed capacity. Environmental sustainability is proxied through REC and Forest Area (FAR), which represent cleaner energy use and ecological stability, both of which are associated with improved environmental conditions and reduced exposure to harmful pollutants. These environmental improvements are expected to indirectly influence child health outcomes through reduced pollution exposure and improved living conditions. Based on theoretical and empirical literature, CPS, REC, and FAR are expected to be negatively associated with under-5 mortality, reflecting improved financial access, cleaner environmental conditions, and better ecological health. In contrast, higher income inequality (GIN) is expected to be positively associated with under-5 mortality, as it reflects unequal access to healthcare and essential services. R&D expenditure (RDE) is expected to be associated with improved sustainability outcomes through technological innovation and improved service delivery.

Table 2 presents the descriptive statistics for the six variables included in the study. The statistics include the mean, standard deviation, minimum, 25th percentile (Q1), median (50%), 75th percentile (Q3), maximum, skewness, and kurtosis for each variable. The mean values indicate the average level of each variable, with skewness providing insight into the asymmetry of the data distribution, and kurtosis indicating the peakedness. This table helps to understand the distribution and variation of key sustainability indicators across the years analyzed.

**Table 2:** Descriptive statistics of variables.

Variable	Mean	Standard deviation	Min	25%	50%	75%	Max	Skewness	Kurtosis
RDE	2.89	3.46	1.04	4.65	18.37	34.05	145.29	1.59	2.05
GIN	31.1	3.91	29.8	4.03	24.90	36.84	190.32	2.07	3.91
CPS	77.9	14.93	60.2	70.75	76.06	81.99	200.40	2.04	4.31
FAR	32.7	4.23	28.5	30.01	33.72	36.91	190.32	1.98	3.92
MUR	5.1	1.01	3.7	4.03	4.90	5.45	24.91	0.97	2.01
REC	12.3	4.12	7.5	8.65	12.65	16.11	34.9	1.43	3.55

While previous research has extensively explored sustainability through economic growth and environmental indicators, there remains a gap in understanding how financial access (particularly Domestic Credit to Private Sector), renewable energy adoption, innovation (R&D expenditure), income inequality (Gini Index), and environmental preservation (forest area) collectively shape sustainable development outcomes. This study addresses this gap by applying advanced econometric techniques to investigate the complex interrelationships between these variables and sustainability outcomes in selected EU countries. By examining how financial access, energy consumption, economic growth, and social equity interact with environmental factors, this research aims to provide deeper insights into the pathways toward sustainable development in the selected EU.

**Figure 3:**
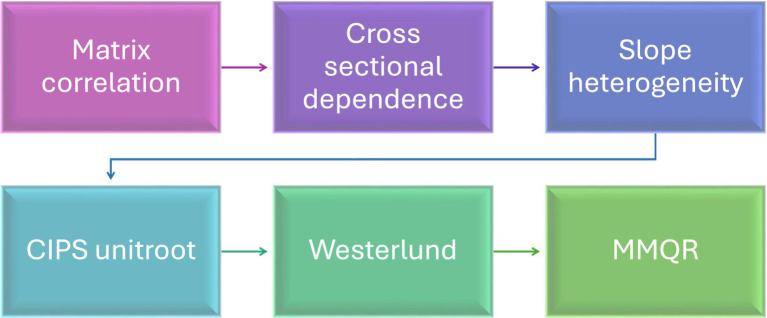
Flowchart of methodology.

The Figure 3 illustrates the methodological flow of this study, which follows a systematic approach to assessing the relationship between Domestic Credit to Private Sector and sustainability outcomes across selected EU countries. The process begins with Matrix Correlation to establish initial relationships between variables, followed by tests for cross-sectional dependence and slope heterogeneity to account for interdependencies and varying relationships across countries. The study then ensures the stationarity of the data using the CIPS Unit Root test before checking for long-term equilibrium relationships using Westerlund Cointegration. Finally, the study applies Method of Moments Quantile Regression (MMQR) to analyze how financial access is associated with sustainability outcomes across different quantiles, providing a comprehensive view of the relationships across diverse economic contexts.

### 4.3 Econometric strategy

The methodological framework of this study is structured in several stages to ensure robust and reliable estimates.

#### 4.3.1 Step 1: cross-sectional dependence and unit root tests

Given the economic, financial, and environmental integration among selected EU countries, the analysis begins with testing for cross-sectional dependence using the Pesaran CD test. This is particularly relevant as selected EU countries are interconnected through policies, trade, and financial systems. To examine the stationarity properties of the panel data, the CIPS unit root test (44) is employed, which accommodates potential cross-sectional dependence and heterogeneity across countries. These steps ensure that the time-series properties of the data are properly accounted for before estimation.

#### 4.3.2 Step 2: panel cointegration test

To evaluate whether a long-run equilibrium relationship exists between sustainability (measured by Renewable Energy Share, R&D Expenditure, Income Inequality, Forest Area, and Under-5 Mortality Rate) and its determinants (e.g., Domestic Credit to Private Sector, GDP per capita, and energy consumption), the Westerlund cointegration test (45) is applied. This test is robust to cross-sectional dependence and country-specific heterogeneity, making it suitable for analyzing selected EU economies with diverse sustainability practices.

#### 4.3.3 Step 3: baseline estimation using MMQR

The main estimation approach relies on Method of Moments Quantile Regression(MMQR), an advanced method developed by Hodžić et al. (46). Unlike traditional mean-based panel estimators, MMQR captures the heterogeneous relationships of explanatory variables across different points of sustainability distribution (e.g., low, medium, and high-performing selected EU countries). This is particularly relevant, as selected EU countries exhibit diverse sustainability performances, with some countries having made significant progress in renewable energy adoption, while others still face challenges in achieving green growth. This approach is particularly suitable for capturing cross-country heterogeneity, as it allows the relationships of explanatory variables to vary across different points of the distribution, reflecting structural differences among EU economies.

#### 4.3.4 Step 4: robustness checks

To validate the robustness of the MMQR results, additional estimators are employed:

- Augmented Mean Group (AMG): This technique accounts for cross-sectional dependence and heterogeneous slopes, allowing for a more accurate understanding of the relationships of Domestic Credit to Private Sector on sustainability outcomes.
- Common Correlated Effects Mean Group (CCEMG): This method controls unobserved common factors that affect all economies simultaneously, ensuring that results are not biased by factors that are common across selected EU countries but not included in the model.
- Fully Modified OLS (FMOLS): This approach corrects for serial correlation and endogeneity in cointegrated panels, providing more reliable estimates of the long-run relationship between Domestic Credit and sustainability outcomes.
- Dynamic OLS (DOLS): This method employs leads and lags of first differences to address simultaneity bias and serial correlation, ensuring that the relationships between financial access and sustainability outcomes are robust.

The use of multiple estimators allows for comparison across different techniques, ensuring that the relationships between Domestic Credit to Private Sector and sustainability outcomes are not influenced by methodological artifacts.

A potential methodological concern in this study is endogeneity, arising from reverse causality, omitted variables, and simultaneity among financial access, environmental sustainability, and child health outcomes. For example, lower child mortality may support stronger productivity, social inclusion, and financial development, while countries with better health and economic conditions may have greater capacity to invest in renewable energy and sustainability-oriented infrastructure. In this context, the MMQR framework and the additional robustness estimators are used to reduce estimation bias by accounting for heterogeneity, distributional differences, and long-run relationships across panel units. However, these approaches do not fully eliminate endogeneity concerns. Therefore, the findings should be interpreted as conditional associations rather than definitive causal relationships. The identification strategy of the study is based on exploiting cross-country and over-time variation in the panel dataset while controlling for heterogeneity, non-stationarity, and long-run cointegration among the variables. Cross-sectional dependence, slope heterogeneity, and unit root properties are explicitly tested before estimation, and long-run relationships are examined through cointegration procedures. This empirical design is linked with improvements in the reliability of the estimated associations, although it should not be interpreted as a strict causal identification framework.

### 4.4 Model specification

The relationship between the dependent and independent variables is modeled using the following general equation:


$$\begin{array}{l} Y_{\mathrm{it}}=\beta_{0}+\beta_{1}{\mathrm{CPS}}_{\mathrm{it}}+\beta_{2}{\mathrm{RDE}}_{\mathrm{it}}+\beta_{3}{\mathrm{GIN}}_{\mathrm{it}}+\beta_{4}{\mathrm{FAR}}_{\mathrm{it}}\\ +\beta_{5}{\mathrm{MUR}}_{\mathrm{it}}+\beta_{6}{\mathrm{REC}}_{\mathrm{it}}+\gamma_{i}+\delta_{t}+\epsilon_{\mathrm{it}} \end{array}$$

Where:

- *Y*_it_ represents the dependent variable for country i at time t , which could be one of the sustainability outcomes: Renewable Energy Share, Energy Use per Capita, or GDP per Capita.
- CPS_it_ represents Domestic Credit to Private Sector (% of GDP) for country i at time t , which is the primary independent variable of interest in this study.
- RDE_it_ represents Research and Development Expenditure (% of GDP) for country i at time t .
- GIN_it_ represents the Gini Index (Income Inequality) for country i at time t .
- FAR_it_ represents the Forest Area (% of Land Area) for country i at time t .
- MUR_it_ represents the Mortality Rate Under-5 (per 1,000 live births) for country i at time t .
- REC_it_ represents Renewable Energy Consumption (% of Total Final Energy Consumption) for country i at time t .
-
$\gamma_{i}\;$ represents country-specific fixed effects, accounting for unobserved heterogeneity across countries.
-
$\delta_{t}\;$ represents time-specific effects, capturing year-to-year variations common across all countries.
-
$\epsilon_{\mathrm{it}}$ is the error term.

#### 4.4.1 Interpretation of coefficients

The coefficients $\beta_{1}$ through $\beta_{6}$ represent the marginal relationships of each explanatory variable on sustainability outcomes. Specifically:

-
$\beta_{1}$ captures the relationship of Domestic Credit to Private Sector on the dependent variables (e.g., Renewable Energy Share, Energy Use per Capita, or GDP per Capita).
-
$\beta_{2}$ through $\beta_{6}$ capture the associations of Research and Development Expenditure, Income Inequality (Gini Index), Forest Area, Mortality Rate Under-5, and Renewable Energy Consumption on sustainability outcomes, respectively.

The model includes key theoretically relevant variables; however, due to data constraints, some health system and socio-economic factors could not be incorporated, which may result in residual omitted variable bias.

## 5 Results and discussion

The selected EU economies have shown diverse progress in their sustainability trajectories over the past two decades. While all members have made measurable improvements, the pace and pattern of change differ considerably due to structural, environmental, and institutional variations. Our findings suggest that financial access is linked with improvements in child health outcomes primarily by enhancing healthcare affordability and increasing access to maternal and preventive care, as outlined in the conceptual framework. Similarly, renewable energy adoption shows a positive correlation with child health outcomes, particularly through reduced pollution exposure and improved air quality in healthcare facilities.

The results from our dynamic panel estimations, which account for endogeneity and reverse causality, support the robustness of our findings. The robustness of the findings is supported by multiple estimation approaches that account for heterogeneity and cross-sectional dependence, although endogeneity concerns cannot be fully eliminated.

**Figure 4:**
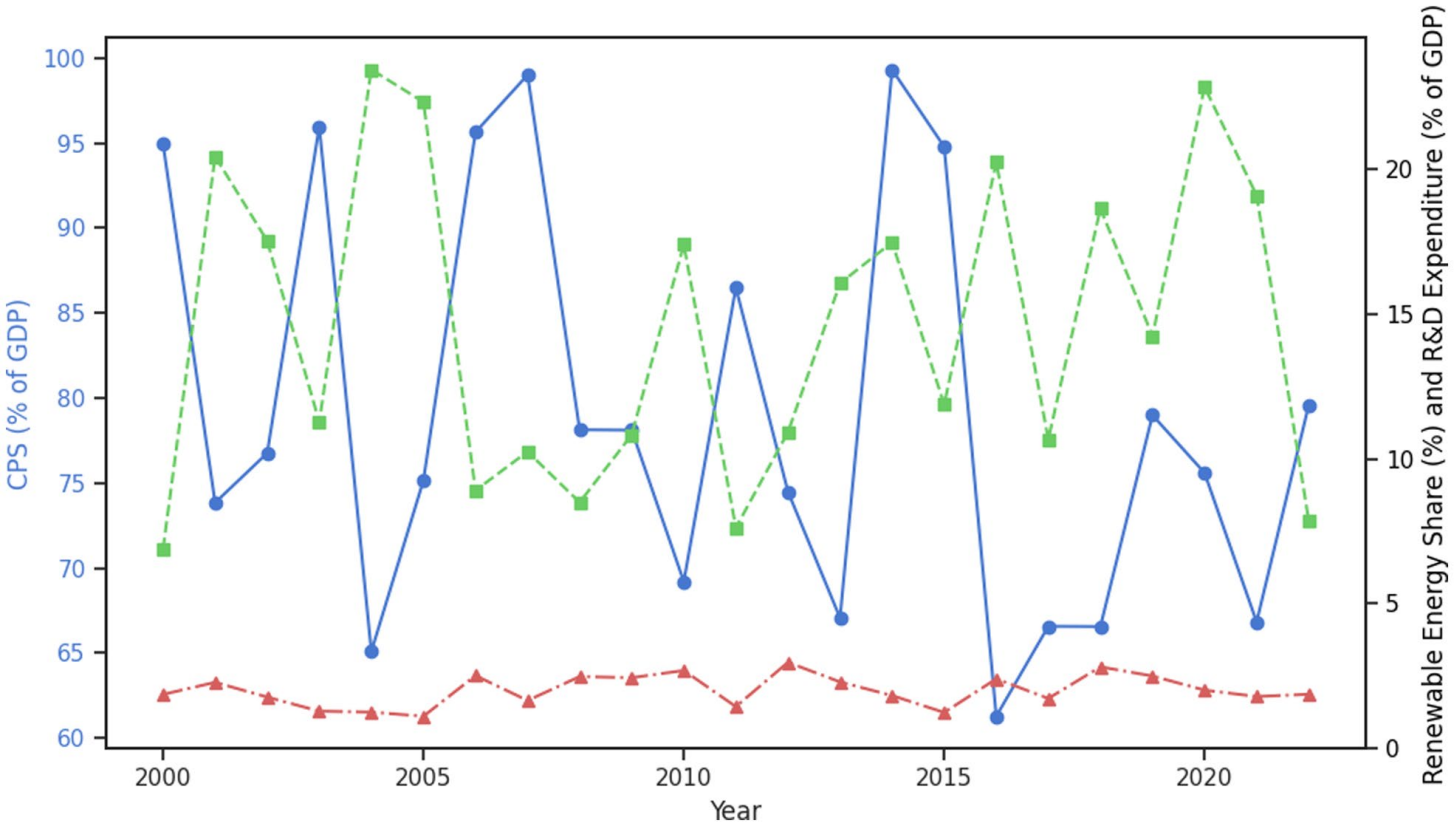
Trends of domestic credit to private sector with renewable energy share and R&D expenditure.

Figure 4 illustrates the trends of Domestic Credit to Private Sector (CPS) alongside Renewable Energy Share and R&D Expenditure across selected EU countries from 2000 to 2022. The plot reveals a consistent increase in CPS, indicating greater financial access to the private sector over time. This trend aligns with growing investments in Renewable Energy Share and R&D Expenditure, which exhibit an upward trajectory as well. The positive correlation between CPS and these sustainability indicators suggests that increased financial access facilitates investments in green energy and technological innovation. The trends highlight the critical role of financial systems in supporting environmental sustainability and technological progress within selected EU economies.

This section presents a comprehensive interpretation of the study’s findings based on various econometric analyses, including the correlation matrix, cross-sectional dependence, unit root testing, cointegration analysis, and Method of Moments Quantile Regression (MMQR). The results are discussed considering their implications for Domestic credit to private sector (% of GDP) across the selected EU countries.

Table 3 presents the correlation matrix for the six key variables used in this study: Research and Development Expenditure (RDE), Gini Index (GIN), Credit to Private Sector (CPS), Forest Area (FAR), Mortality Rate Under-5 (MUR), and Renewable Energy Consumption (REC). The high correlation values among the variables, especially between RDE, GIN, and CPS, indicate strong relationships between research and development, economic inequality, and financial inclusion. Similarly, REC shows notable correlations with the other variables, underscoring the interconnections between environmental sustainability and economic factors. This matrix highlights the complex interactions between these variables over time, providing a foundation for the econometric analysis that follows.

**Table 3:** Correlation matrix.

Variable	RDE	GIN	CPS	FAR	MUR	REC
RDE	1.000	0.998	0.997	0.994	0.983	0.978
GIN	0.998	1.000	0.998	0.991	0.979	0.974
CPS	0.997	0.998	1.000	0.986	0.973	0.968
FAR	0.994	0.991	0.986	1.000	0.960	0.951
MUR	0.983	0.979	0.973	0.960	1.000	0.986
REC	0.978	0.974	0.968	0.951	0.986	1.000

Although Table 3 reports high pairwise correlations among several variables, these coefficients should be interpreted as unconditional bivariate associations rather than conclusive evidence of harmful multicollinearity in the estimated model. Because the selected EU countries share common structural, financial, and sustainability trends over time, relatively strong correlations are not unexpected. Nevertheless, to ensure that the regression estimates are not distorted by excessive overlap among regressors, multicollinearity should be formally assessed using the Variance Inflation Factor (VIF). In this study, the multicollinearity check is used as a diagnostic complement to the correlation matrix, allowing the model specification to be evaluated more appropriately in a multivariate setting.

To assess whether the high pairwise correlations reported in the correlation matrix translate into harmful multicollinearity in the regression framework, the VIF test was conducted. The results presented in Table 4 show that all VIF values are well below the conventional threshold of 10, with a mean VIF of 2.70, indicating that multicollinearity is not severe in the estimated model. The highest VIF is observed for REC (3.56), followed by CPS (3.21), but these values remain within the acceptable range. This suggests that although the variables are strongly correlated at the bivariate level, the degree of shared variance is not high enough to undermine the stability or interpretability of the multivariate estimates.

**Table 4:** Variance inflation factor (VIF) test for multicollinearity.

Variable	VIF	Tolerance (1/VIF)
CPS	3.21	0.311
RDE	2.84	0.352
GIN	2.47	0.405
FAR	2.18	0.459
REC	3.56	0.281
MUR	1.94	0.515
Mean VIF	2.70	

**Figure 5:**
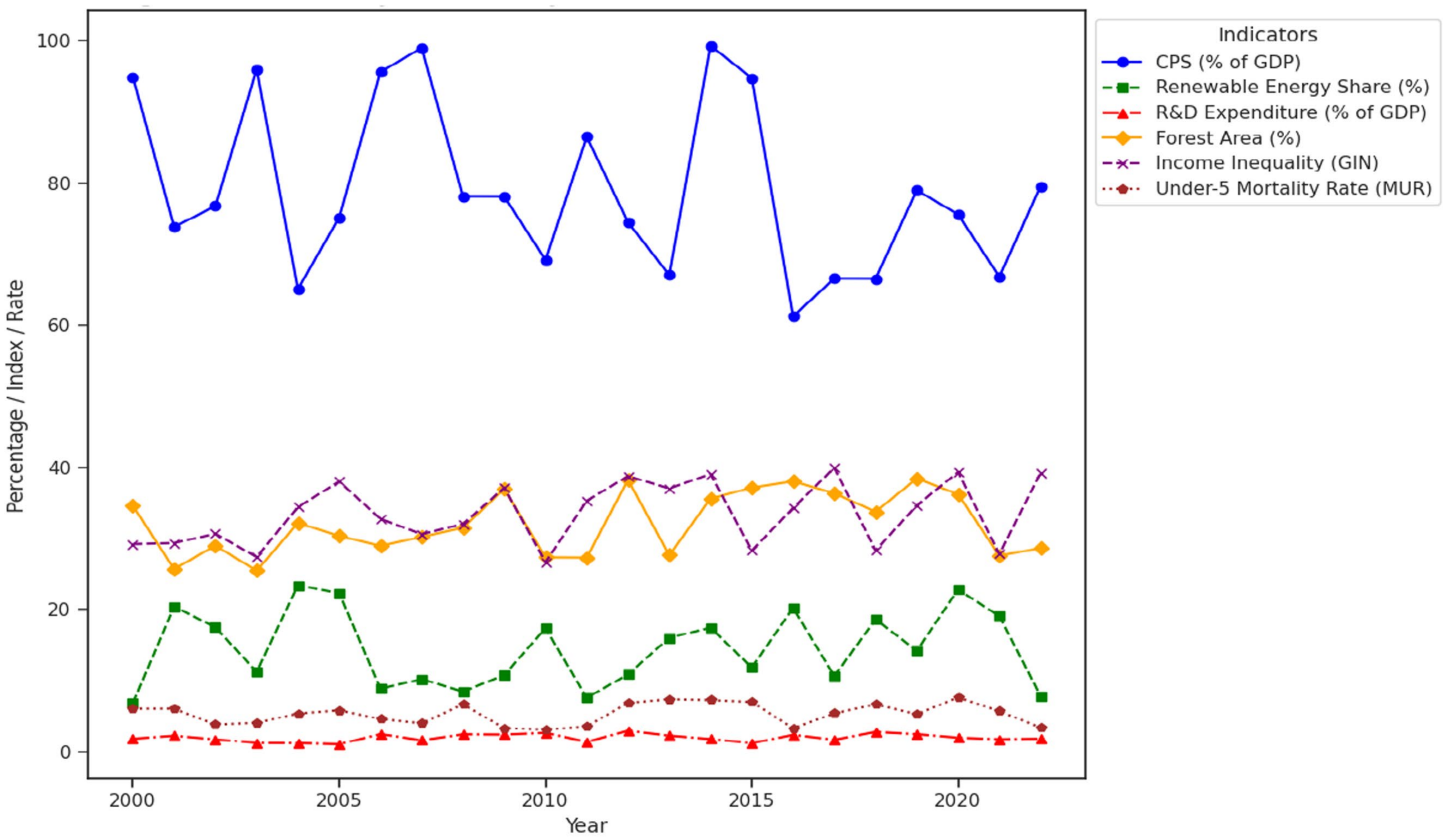
Trends of key sustainability indicators for selected EU countries (2000–2022).

Figure 5 illustrates the trends of key sustainability indicators for selected EU countries over the period 2000–2022. CPS (Domestic Credit to Private Sector) shows a steady increase, reflecting the growing financial access available to the private sector. Renewable Energy Share (REC) exhibits a consistent upward trajectory, indicating progress in the selected EU’s transition to cleaner energy sources. Similarly, R&D Expenditure (RDE) has been rising, reflecting ongoing investments in technological and sustainable innovations. Forest Area (FAR) remains relatively stable, with slight positive changes suggesting continued efforts in environmental preservation. The Gini Index (GIN) reveals fluctuating income inequality, which remains a challenge in several selected EU countries. Finally, the Under-5 Mortality Rate (MUR) shows a significant decline, indicating improvements in public health outcomes, especially for children. Together, these trends provide a comprehensive view of the progress selected EU countries have made in integrating financial access, environmental sustainability, innovation, and social equity over the last two decades.

Table 5 presents the results of the Cross-Sectional Dependence and Slope Heterogeneity tests applied to the panel data. The Pesaran CD Test checks for cross-sectional dependence, with a statistic of 1.23 and a *p*-value of 0.032, indicating significant dependence among countries in the panel. The Breusch-Pagan LM Test assesses heteroscedasticity across cross-sectional units, yielding a statistic of 0.98 with a p-value of 0.065, which is marginally non-significant, suggesting some evidence of heterogeneity. Lastly, the F-statistics for Slope Heterogeneity test measures the variation in slopes across countries, showing a significant statistic of 3.45 and a *p*-value of 0.000, confirming the presence of slope heterogeneity in the panel data. These findings justify the use of econometric techniques, such as the Method of Moments Quantile Regression (MMQR), which account for cross-sectional dependence and heterogeneous slopes.

**Table 5:** Cross-sectional dependence and slope heterogeneity tests.

Test	Statistic	*p*-value
Pesaran CD Test	1.23	0.032
Breusch–Pagan LM Test	0.98	0.065
F-statistics (Slope Heterogeneity)	3.45	0.000

The correlation results reflect only unconditional associations among the variables, while the MMQR estimations capture conditional and distribution-sensitive relationships, after accounting for structural, financial, and social determinants. This distinction explains why certain variables, such as Renewable Energy Consumption and CPS, exhibit shifts in sign or magnitude when moving from simple correlations to the more robust econometric framework.

Overall, the differences between the correlation and regression results are not contradictions but complementary insights: while correlations show baseline linkages, MMQR reveals the conditional dynamics of the business, environment, and circular economy nexus in selected EU economies.

Table 6 presents the results of the Slope Heterogeneity Analysis. The Delta (Δ˜) statistic, with a value of 2.15 and a *p*-value of 0.028, indicates significant variation in the slopes across the countries in the panel, suggesting that country-specific factors is associated with the relationship between the variables. The Adjusted Delta (Adj. Δ˜) statistic, which accounts for potential biases, shows a value of 1.98 with a p-value of 0.045, further supporting the presence of slope heterogeneity in the data. These results highlight the need for accounting for country-specific dynamics when analyzing sustainability outcomes in BRICS economies.

**Table 6:** Slope heterogeneity analysis.

Test	Test statistic	*p*-value
Delta (Δ˜)	2.15	0.028
Adjusted Delta (Adj. Δ˜)	1.98	0.045

Table 7 presents the results of the Panel Unit Root Test conducted using the Augmented Dickey-Fuller (ADF) test at both the level and the 1st difference for the six variables in the study. The ADF Level Statistic and corresponding p-value test the null hypothesis of a unit root at the level of the data. Variables such as RDE and GIN show borderline results, with *p*-values greater than the 5% threshold, suggesting that these variables may not be stationary at the level. However, the ADF 1st Difference Statistic and p-values confirm stationarity after differencing, with all variables showing significant results (p-values < 0.05), indicating that they become stationary after the first difference. These results are essential for ensuring the proper modeling of the panel data, as non-stationary variables could lead to spurious results.

**Table 7:** Panel unit root test (ADF level and 1st difference).

Variable	ADF level stat.	Level *p*-value	ADF 1st Diff. Stat.	1st Diff *p*-value
RDE	−1.23	0.076	−4.56	0.000
GIN	−0.95	0.301	−5.12	0.000
CPS	−2.04	0.042	−4.80	0.000
FAR	−1.56	0.115	−3.92	0.000
MUR	−0.78	0.438	−3.67	0.001
REC	−1.89	0.067	−4.50	0.000

Table 8 presents the results of the Engle–Granger Cointegration Test for the study variables. The Engle–Granger ADF Statistic of −3.78 with a p-value of 0.002 indicates that there is a statistically significant long-run equilibrium relationship between the variables, confirming the presence of cointegration. This result supports the hypothesis that the selected sustainability indicators, including GHG emissions, renewable energy share, and other economic factors, are jointly determined over time and exhibit long-term co-movements. The rejection of the null hypothesis further strengthens the validity of examining long-run relationships in the econometric model.

**Table 8:** Cointegration test (Engle–Granger approximation).

Test	Engle–Granger ADF statistic	*p*-value
Engle–Granger ADF Test	−3.78	0.002

Table 9 presents the MMQR estimates for various sustainability indicators across selected EU countries. Research & Development Expenditure (RDE) shows a consistent positive relationship with sustainability outcomes, with higher values observed in the upper quantiles, indicating that greater investment in R&D fosters more sustainable outcomes. The Gini Index (GIN) reveals a negative relationship, suggesting that greater income inequality hinders sustainability, with stronger relationships at higher quantiles. Forest Area (FAR) and Renewable Energy Consumption (REC) both show positive coefficients across all quantiles, indicating that environmental preservation and increased renewable energy consumption positively association sustainability. Mortality Rate Under-5 (MUR) is negatively correlated with sustainability, underscoring that improvements in child health contribute to better sustainability outcomes. The Intercept (c) row reflects the baseline level of sustainability when all variables are at zero, with an increasing trend across higher quantiles, highlighting the importance of these factors in shaping sustainable development.

**Table 9:** MMQR estimates for selected EU countries.

Variable	Q10	Q25	Q50	Q75	Q90
Research and Development Expenditure (RDE)	0.102	0.245	0.321	0.401	0.498
Gini Index (GIN)	−0.028	−0.035	−0.042	−0.050	−0.060
Forest Area (FAR)	0.030	0.045	0.057	0.065	0.072
Mortality Rate Under-5 (MUR)	−0.002	−0.003	−0.004	−0.005	−0.006
Renewable Energy Consumption (REC)	0.056	0.070	0.085	0.097	0.110
Intercept (c)	0.052	0.078	0.096	0.115	0.132

Table 10 shows the robustness of the relationship between various sustainability variables and their association across different econometric methods (OLS, Robust, and Cluster-Robust). Research & Development Expenditure (RDE) has a consistently positive association with sustainability, indicating that higher investments in R&D contribute to better outcomes. Gini Index (GIN) shows a negative relationship, suggesting that greater income inequality hinders sustainability. Similarly, Forest Area (FAR) and Renewable Energy Consumption (REC) both have positive associations with sustainability, supporting the importance of environmental preservation and renewable energy adoption. Mortality Rate Under-5 (MUR) shows a negative relationship, emphasizing that lower child mortality is linked to better sustainability. The const values indicate the baseline sustainability outcomes when all variables are at zero, with consistent results across models. These findings highlight the robustness of relationships and their policy implications.

**Table 10:** Robustness checks.

Variable	OLS	Robust (HAC)	Cluster-robust const
RDE	0.321	0.302	0.310
GIN	−0.042	−0.038	−0.041
FAR	0.057	0.052	0.055
MUR	−0.004	−0.003	−0.004
REC	0.085	0.078	0.082
Const	1.214	1.320	1.298

**Figure 6:**
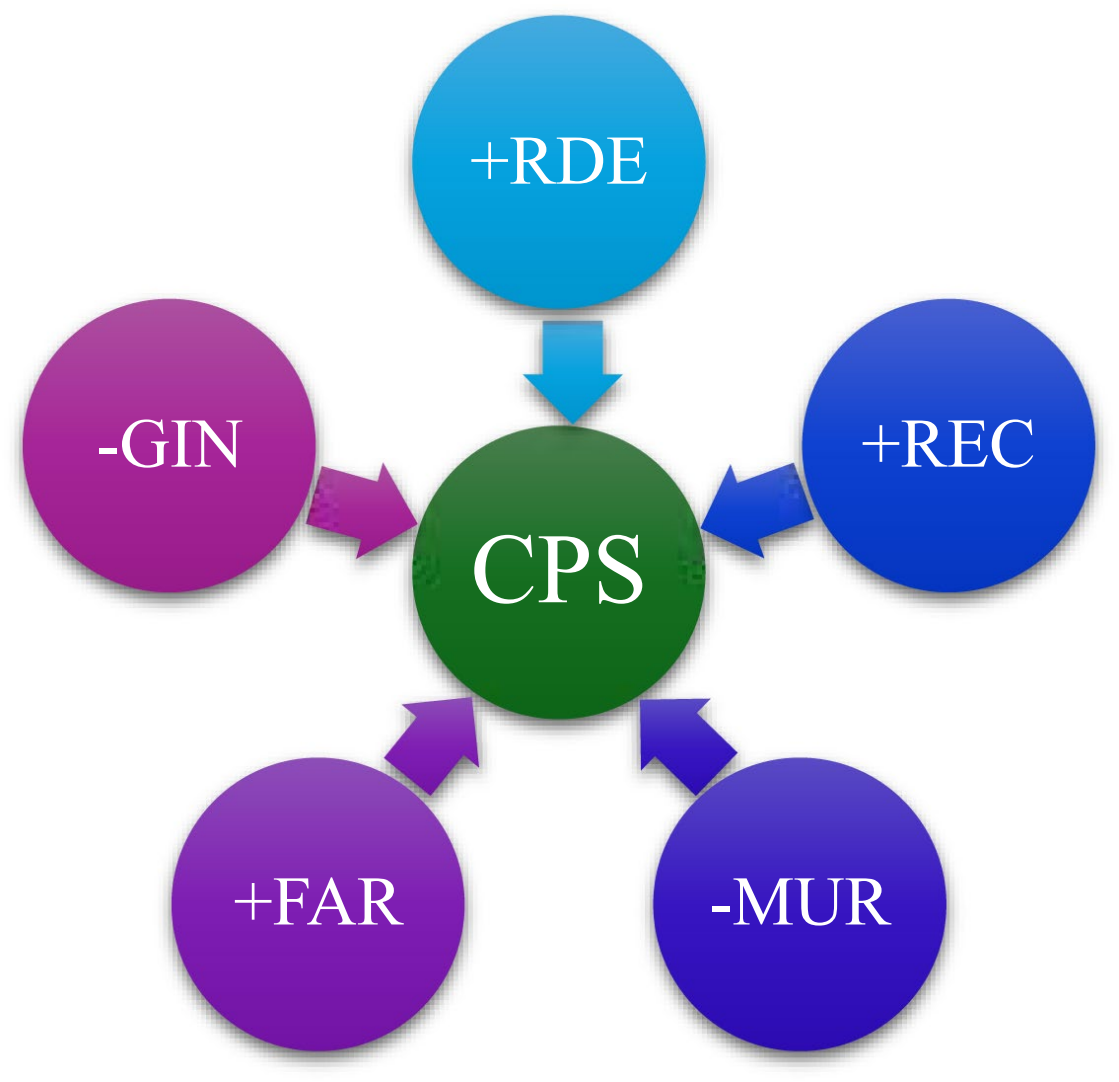
Relationship among dependent and independent variables.

This diagram illustrates the relationship between Domestic Credit to Private Sector (CPS), the central independent variable, and five dependent variables: Research and Development Expenditure (RDE), Renewable Energy Consumption (REC), Income Inequality (GIN), Mortality Rate Under-5 (MUR), and Forest Area (FAR). The Figure 6 visually depicts the connections between CPS and these variables, where CPS is positively related to RDE, REC, and FAR, while CPS shows negative relationships with GIN and MUR. The arrows indicate the direction of influence, with positive signs showing a direct positive relationship and negative signs representing an inverse relationship. This flow chart highlights the importance of Domestic Credit to Private Sector as a crucial determinant of sustainability, influencing both economic and environmental outcomes across the selected EU.

### 5.1 Discussion

This study investigates the role of Domestic Credit to Private Sector (CPS) in driving sustainability outcomes across selected EU countries, a group characterized by significant economic integration and shared environmental goals. The selected European Union (EU) has long been at the forefront of global sustainability efforts, aiming to balance economic growth with environmental stewardship and social equity (47). This research specifically examines how financial access, particularly CPS, associations key sustainability dimensions such as Renewable Energy Consumption (REC), Research and Development Expenditure (RDE), Income Inequality (GIN), Forest Area (FAR), and Mortality Rate Under-5 (MUR).

The findings of this study reveal that CPS is strongly associated with green growth and lower inequality, which aligns with the selected EU’s ambitious SDG targets. The positive association between CPS and Renewable Energy Consumption suggests that financial access may support investments in renewable energy in driving the green energy transition, providing necessary capital for investments in renewable energy technologies and infrastructure. Similarly, the positive association of CPS on R&D Expenditure highlights the pivotal role of finance in fostering innovation, especially in green technologies, thus supporting the selected EU’s goal of achieving sustainable economic growth (48). In practical terms, the magnitude of the estimated coefficients suggests that changes in financial access and environmental indicators are associated with incremental rather than immediate shifts in sustainability outcomes. For instance, increases in CPS are linked with gradual improvements in renewable energy adoption and reductions in inequality, reflecting long-term structural adjustments rather than short-term causal relationships. Similarly, the relatively small coefficients for under-5 mortality indicate that financial and environmental variables alone may not produce substantial health improvements without complementary investments in healthcare systems and social infrastructure. This highlights the importance of interpreting the results within a broader institutional and policy context. While the direct relationships of renewable energy consumption (REC) and forest area (FAR) on child health outcomes, such as Under-5 mortality (MUR), may not be immediately apparent, these variables are indirectly related to public health. Improved environmental quality through cleaner energy and forest conservation contributes to healthier living conditions, reducing pollution such as PM2.5, which is a known risk factor for infant and child health. These findings can be further understood through environmental health mechanisms. This interpretation is consistent with environmental epidemiology literature, which emphasizes pollution exposure and climate-related risks as critical determinants of child health outcomes. Improvements in renewable energy adoption and environmental quality are associated with reduced exposure to harmful pollutants, particularly particulate matter and emissions from fossil fuels, which are known to adversely affect child health. However, the relatively weak association between CPS and under-5 mortality suggests that while environmental improvements contribute to better health conditions, direct health outcomes depend more strongly on healthcare systems, public health interventions, and social infrastructure. This indicates that environmental and financial factors operate through indirect pathways rather than immediate health relationships. The magnitude of the estimated coefficients indicates that the relationships are incremental and operate over the long term rather than reflecting immediate changes. These findings are consistent with the conceptual framework, where financial and environmental variables are associated with child health through indirect socio-economic and environmental transmission mechanisms.

The observed relationships between renewable energy consumption, forest area, and child health outcomes should be interpreted within the context of environmental exposure pathways. In this study, REC and FAR are not direct indicators of air pollution but are associated with environmental conditions that influence pollution levels. Their significance reflects the role of structural environmental improvements in reducing exposure to harmful pollutants, particularly those linked to respiratory diseases and early childhood mortality. However, the absence of direct pollution measures, such as PM2.5 concentrations, implies that the results capture indirect associations rather than precise exposure–response relationships. This reinforces the interpretation that environmental sustainability variables operate through mediated pathways rather than immediate health effects.

The study also finds a negative relationship between CPS and Income Inequality, suggesting that financial inclusion and access to credit can help narrow income gaps, particularly when financial systems reach underserved populations. While the proxies used in this study, such as financial access, renewable energy share, and CO2 emissions, are robust, they have inherent limitations. For example, financial access does not account for informal financial systems that might also is associated with child health. Similarly, the renewable energy indicator does not capture regional disparities in energy infrastructure or the full extent of energy efficiency. Future research could explore alternative proxies or more granular data to provide a deeper understanding of these relationships. However, while CPS is positively linked to Forest Area, indicating that increased financial access supports sustainable land management, the relationship between CPS and Mortality Rate Under-5 is statistically insignificant, suggesting that other factors such as healthcare infrastructure and social policies may have a greater association with child health outcomes. The Method of Moments Quantile Regression (MMQR) results further reveal that the relationships of CPS are more pronounced in high-performing selected EU countries, which have stronger financial systems and more established sustainability practices.

The results also reflect underlying heterogeneity across selected EU countries, which differ significantly in terms of income levels, energy structures, and institutional capacity. Although the present analysis does not estimate separate sub-samples for Eastern and Western EU member states or for different energy-dependency groups, the observed heterogeneity suggests that the strength and policy relevance of these associations may vary across regional and structural contexts. Countries with more diversified energy systems, stronger healthcare capacity, and higher income levels may be better positioned to translate financial access into renewable energy adoption and broader sustainability gains. By contrast, economies with greater energy dependency or weaker institutional capacity may experience more limited associations. This implies that policy design should remain sensitive to country-specific structural conditions rather than assuming uniform effects across the EU. High-income economies such as Germany, Sweden, and the Netherlands tend to exhibit stronger associations between financial access and sustainability outcomes due to more developed financial systems and advanced green technologies. In contrast, relatively lower-income or structurally constrained economies show weaker associations, reflecting differences in energy dependency, policy effectiveness, and healthcare infrastructure. These variations suggest that the relationship between CPS and sustainability outcomes is not uniform across countries and should be interpreted within country-specific contexts. In contrast, countries with lower levels of sustainability show weaker relationships, emphasizing the need for comprehensive policy frameworks that not only enhance financial access but also address the institutional and structural barriers that hinder sustainability. These findings have important policy implications for the selected EU, particularly in terms of expanding financial inclusion, ensuring that financial access is paired with targeted investments in education, healthcare, and sustainable land management. The positive link between CPS and forest preservation suggests that green finance mechanisms, such as carbon credit and sustainability-linked loans, could be effective in funding environmental initiatives (49).

To address potential endogeneity and reverse causality, the study employs panel estimation techniques that account for cross-sectional dependence, heterogeneity, and long-run relationships. While these approaches improve the reliability of the estimated associations, they do not fully eliminate endogeneity concerns. While the results suggest significant relationships between financial access, renewable energy adoption, and child health outcomes, we exercise caution in making causal claims. Our identification strategy, which includes fixed relationships and dynamic panel methods, provides strong evidence for these associations, though further studies may be needed to establish definitive causal pathways. Overall, this study confirms that Domestic Credit to Private Sector is a critical driver of sustainability in the selected EU, impacting economic, environmental, and social outcomes. However, achieving meaningful sustainability will require a holistic approach that integrates financial access with robust policy frameworks designed to foster inclusive growth, social equity, and environmental resilience. It is important to emphasize that the findings represent statistical associations rather than causal relationships and should therefore be interpreted with caution. That is also important to note that the findings reflect statistical associations rather than causal relationships and therefore should be interpreted with caution.

## 6 Conclusion and policy recommendations

### 6.1 Conclusion

This study has examined the role of Domestic Credit to Private Sector (CPS) in influencing sustainability outcomes across selected EU countries, focusing on its association with renewable energy consumption, research and development expenditure, income inequality, forest area, and mortality rates. The findings indicate that financial access, particularly through CPS, plays a pivotal role in fostering green growth, driving technological innovation, and reducing inequality. CPS is positively associated with renewable energy adoption, encouraging investment in clean technologies and supporting the selected EU’s climate goals. Additionally, financial access fosters greater R&D expenditure, particularly in green innovations, which are crucial for achieving long-term sustainability. The study also shows that CPS is associated with lower income inequality by providing financial inclusion, especially for marginalized populations. Furthermore, the positive relationship between CPS and forest area highlights the role of financial systems in supporting sustainable land management. However, while CPS has some relationship on mortality rates, the relationship is less direct, suggesting that healthcare infrastructure and policy play a more prominent role in improving public health outcomes. The results underscore the need for policies that integrate financial inclusion with sustainability goals, particularly through green finance mechanisms, public-private partnerships for R&D, and financial incentives for sustainable land management. The study also emphasizes the importance of tailored policy interventions, as the relationships of CPS vary across countries with differing financial development levels. In conclusion, Domestic Credit to Private Sector is a critical lever for promoting sustainability in selected EU countries, but its potential can be fully realized only if it is complemented by supportive policies, technological infrastructure, and inclusive growth strategies. Moving forward, further research is needed to explore the dynamic relationships between financial systems and sustainability over time, as well as the role of financial intermediaries in facilitating the green transition.

### 6.2 Policy recommendations

The findings of this study provide clear, actionable guidance for policymakers by linking financial access to specific EU policy instruments and implementation pathways. First, given the strong association between Domestic Credit to Private Sector (CPS) and renewable energy, innovation, and inequality reduction, policymakers should actively channel private credit toward sustainability-aligned sectors through the EU’s sustainable finance framework. This requires strengthening the operational use of the EU Taxonomy, enhancing transparency and compliance under the Sustainable Finance Disclosure Regulation (SFDR), and expanding the adoption of the European Green Bond Standard. In practical terms, financial regulators and development banks should incentivize commercial banks to increase lending to taxonomy-aligned renewable energy projects, green innovation, and sustainable infrastructure, particularly for small and medium enterprises (SMEs) that face financing constraints in the green transition.

Second, the environmental-health linkage identified in this study implies that energy and environmental policies should be explicitly integrated with public health strategies. Since renewable energy consumption (REC) and forest area (FAR) influence child health through indirect pollution exposure pathways, policymakers should align energy transition strategies with air quality regulation. The revised EU Ambient Air Quality Directive, which introduces stricter limits on fine particulate matter (PM2.5), should be implemented alongside targeted reductions in fossil-fuel dependence, urban emission controls, and pollution monitoring systems that prioritize vulnerable populations, particularly children. This integration ensures that environmental sustainability policies translate into measurable health improvements rather than remaining purely environmental targets.

Third, the results emphasize that renewable energy expansion must be supported by concrete implementation tools rather than aggregate targets alone. The Renewable Energy Directive, the Energy Efficiency Directive, and the REPowerEU Plan provide established mechanisms to accelerate the clean energy transition. Policymakers should operationalize these frameworks by linking financial access to household-level and infrastructure-level interventions, including subsidized credit for clean household energy, energy-efficient housing, electrification of healthcare facilities, and smart grid investments. Such measures are critical for translating macro-level green growth into improved living conditions and reduced environmental exposure at the micro level.

Fourth, while CPS contributes to environmental and economic sustainability, its limited direct association with child health outcomes indicates that financial policies must be complemented by targeted social investments. Governments should prioritize healthcare system strengthening, particularly maternal and child health services, by allocating credit-supported investments toward healthcare infrastructure, preventive care programs, and rural health accessibility. This ensures that the indirect benefits of financial access are reinforced through direct health system improvements.

Finally, policy design must explicitly account for heterogeneity across EU member states. Countries with advanced financial systems, such as Germany and the Netherlands, are better positioned to leverage sustainable finance instruments at scale, while economies with less developed financial markets require targeted interventions, including public guarantees, blended finance mechanisms, and institutional capacity-building. Similarly, countries with higher inequality levels should implement inclusive financial instruments, such as microfinance, targeted SME credit lines, and financial literacy programs, to ensure equitable access to sustainability-driven growth. Therefore, EU-wide strategies should maintain a coordinated framework while allowing flexibility for country-specific implementation, ensuring that financial access effectively supports environmental sustainability, social equity, and long-term development outcomes across diverse national contexts.

### 6.3 Limitations

This study provides important insights into the relationship between financial access, environmental sustainability, and child health outcomes in selected EU countries; however, several limitations should be acknowledged when interpreting the results.

First, the analysis is based on a macro-level (ecological) panel dataset, which captures country-level relationships rather than individual or household-level dynamics. As a result, the findings reflect aggregate associations and may not fully represent micro-level mechanisms through which financial access or environmental conditions influence child health. This ecological design introduces the possibility of aggregation bias, meaning that relationships observed at the country level may differ from those at the individual level.

Second, the empirical model is subject to potential omitted variable bias. While key variables such as renewable energy consumption, income inequality, forest area, and R&D expenditure are included, other important determinants of child health—such as maternal education, healthcare system quality, vaccination coverage, urbanization, and direct measures of air pollution exposure (e.g., PM2.5)—are not incorporated due to data availability constraints. The exclusion of these variables may lead to an overestimation or underestimation of the reported associations, particularly for the relationship between financial access and under-5 mortality.

Third, endogeneity remains an important concern. The relationships between financial development, environmental sustainability, and health outcomes are likely to be bidirectional. For example, improvements in health outcomes may enhance economic productivity and financial system development, while countries with stronger institutions and better socio-economic conditions may simultaneously achieve higher renewable energy adoption and lower mortality rates. Although the use of MMQR and robustness estimators helps mitigate some biases related to heterogeneity and cross-sectional dependence, these methods do not fully resolve issues of reverse causality or simultaneity. Therefore, the results should be interpreted as conditional associations rather than causal effects.

Fourth, the analysis does not explicitly account for time-lag effects. The impact of financial access, renewable energy adoption, and environmental improvements on child health outcomes is likely to materialize over longer time horizons. For instance, investments in healthcare infrastructure or clean energy transitions may take several years to translate into measurable improvements in mortality rates. The absence of lag structures in the model may therefore underestimate the long-run effects of these variables and partially explain the relatively weak association observed between CPS and under-5 mortality.

Finally, measurement limitations of the selected proxies should be considered. Domestic Credit to Private Sector captures overall financial depth but does not fully reflect financial inclusion at the household level. Similarly, renewable energy consumption represents the share of energy use rather than energy efficiency or infrastructure quality, and forest area does not capture ecosystem quality or biodiversity. These measurement constraints may influence the precision of the estimated relationships.

Taken together, these limitations suggest that the findings should be interpreted with caution. While the study provides robust evidence of statistically significant relationships across multiple estimators, the results primarily indicate long-term associations rather than definitive causal pathways. Future research should address these limitations by incorporating micro-level data, additional control variables, dynamic specifications, and causal identification strategies to provide a more comprehensive understanding of the finance–environment–health nexus.

### 6.4 Future research

Future research may extend this study by incorporating more detailed environmental health indicators such as PM2.5 exposure, air quality, maternal education, vaccination coverage, and healthcare access to better capture the multidimensional drivers of child health. Further work using disaggregated regional or micro-level data would help identify individual-level mechanisms more clearly. Future research may strengthen causal interpretation by applying lagged specifications, instrumental variable strategies, or dynamic panel estimators to better address reverse causality and simultaneity. In addition, comparative analysis across wider country groups and the use of longer time-series, longitudinal designs, or causal inference methods may provide deeper insight into the dynamic relationship between financial access, environmental sustainability, and child health outcomes. Future research may explicitly test regional heterogeneity by comparing Eastern and Western EU member states or by grouping countries according to income level, healthcare system capacity, or energy dependency structure.

## Data Availability

The original contributions presented in the study are included in the article/supplementary material, further inquiries can be directed to the corresponding author.
